# Human mesenchymal stem cell therapy promotes retinal ganglion cell survival and target reconnection after optic nerve crush in adult rats

**DOI:** 10.1186/s13287-020-02130-7

**Published:** 2021-01-19

**Authors:** Almir Jordão da Silva-Junior, Louise Alessandra Mesentier-Louro, Gabriel Nascimento-dos-Santos, Leandro Coelho Teixeira-Pinheiro, Juliana F. Vasques, Luiza Chimeli-Ormonde, Victor Bodart-Santos, Luiza Rachel Pinheiro de Carvalho, Marcelo Felippe Santiago, Rosalia Mendez-Otero

**Affiliations:** 1 Instituto de Biofísica Carlos Chagas Filho, Universidade Federal do Rio de Janeiro, Rio de Janeiro, RJ, 21941-902 Brazil; 2 Instituto Nacional de Ciência e Tecnologia em Medicina Regenerativa-REGENERE, Rio de Janeiro, RJ, Brazil; 3 Rede NanoSaúde, Rio de Janeiro, RJ Brazil; 4 Department of Ophthalmology, Stanford University, Palo Alto, CA USA

**Keywords:** Central nervous system, Optic nerve injury, Nerve regeneration, Neuroprotection, Cell therapy, Mesenchymal stem cells

## Abstract

**Background:**

Optic-nerve injury results in impaired transmission of visual signals to central targets and leads to the death of retinal ganglion cells (RGCs) and irreversible vision loss. Therapies with mesenchymal stem cells (MSCs) from different sources have been used experimentally to increase survival and regeneration of RGCs.

**Methods:**

We investigated the efficacy of human umbilical Wharton’s jelly-derived MSCs (hWJ-MSCs) and their extracellular vesicles (EVs) in a rat model of optic nerve crush.

**Results:**

hWJ-MSCs had a sustained neuroprotective effect on RGCs for 14, 60, and 120 days after optic nerve crush. The same effect was obtained using serum-deprived hWJ-MSCs, whereas transplantation of EVs obtained from those cells was ineffective. Treatment with hWJ-MSCs also promoted axonal regeneration along the optic nerve and reinnervation of visual targets 120 days after crush.

**Conclusions:**

The observations showed that this treatment with human-derived MSCs promoted sustained neuroprotection and regeneration of RGCs after optic nerve injury. These findings highlight the possibility to use cell therapy to preserve neurons and to promote axon regeneration, using a reliable source of human MSCs.

## Background

Optic nerve injuries resulting from optic neuropathies, traumas, or tumors are characterized by optic nerve degeneration, resulting in partial to complete loss of vision [1]. These injuries specifically affect the RGCs, whose axons form the optic nerve, and their cell bodies degenerate as a consequence of the injury [2]. For instance, glaucoma, the leading cause of RGC degeneration, affects more than 70 million people worldwide, 8 million of whom suffer irreversible bilateral blindness [3]. No clinical treatment is available to sustain RGC survival and promote their regeneration.

The ongoing search for therapies that promote RGC survival and axonal regeneration has impelled many in vitro and in vivo studies [4]. Intraorbital optic nerve crush is a well-established model that leads to progressive degeneration of RGCs, mostly by apoptosis [5–12]. Although RGCs enter a regenerative state after axonal lesion, by upregulating growth-associated protein 43 (GAP43) and activating transcription factor 3, among other factors [13–15], they do not successfully extend their axons beyond the lesion site [16]. Non-neuronal factors such as glial cells and matrix molecules [17–19] and intrinsic neuronal factors contribute to this regenerative failure [20–22]. Furthermore, optic nerve injury interrupts the connection with axonal targets such as the dorsal lateral geniculate nucleus (dLGN) and the superior colliculus (SC), which results in failure in shuttling trophic factors [3, 23, 24].

Several efforts have been made to enhance the regenerative potential of RGCs. For example, deletion of the phosphatase and tensin homolog (*PTEN*) and the suppressor of cytokine signaling 3 (*Socs3*) genes, individually or simultaneously [11, 25], significantly increased the regenerative capacity of RGCs after optic nerve lesion [26]. Axonal regeneration is further increased when *PTEN* deletion is combined with an inflammatory stimulus, inducing RGC regeneration up to the LGN, the suprachiasmatic nucleus and the SC, resulting in partial recovery of visual responses [10, 27]. However, most of these approaches are based on Cre-recombinase techniques, which are not yet suitable for use as clinical therapies.

Cell therapies have emerged as a more viable alternative clinical approach in recent years. Transplanted cells can respond to signals from the lesion environment, activate neuroprotective and pro-regenerative pathways, and even overcome deleterious effects of inflammation [28, 29]. Previous studies from our group have shown that cell therapy with adult bone marrow-derived mononuclear cells (BMMC) increases RGC survival in an optic nerve crush model for a short time period, and some axons can reach and establish synapses in the SC [8, 30]. Alternatively, our group has shown that mesenchymal stem cells (MSCs) remain in the rat vitreous body for at least 18 weeks, providing prolonged neuroprotection to RGCs [6] and inducing strong axonal regeneration up to their brain targets [31]. Also, MSCs have been shown to protect RGCs in models of ischemia/reperfusion [32] and ocular hypertension [33–35], among others.

Compared to other stem cells, MSCs have several advantages, including a low risk of rejection, and simple isolation and culture [36, 37]. It has been suggested that MSCs act through paracrine mechanisms, either by releasing factors directly or from extracellular vesicles (EVs), membrane-covered structures that include microvesicles and exosomes [38–40]. EVs can transfer proteins, bioactive lipids, RNAs, and microRNAs, which can modulate entire signaling pathways [38, 39].

For future clinical studies, it is important to investigate the effects of human MSCs on RGCs after optic nerve injury. MSCs from human Wharton’s jelly (hWJ-MSCs) have been suggested as a potential source for cell therapies because they are easily obtained and multiplied in vitro [36] and, as recently shown by our group, promote RGC survival in vitro by paracrine mechanisms [41]*.* In the present study, we found that a single intravitreal injection of hWJ-MSCs, cultured in the presence or absence of serum, was neuroprotective, but their EVs were not in the dose used here. Moreover, the neuroprotective effect was sustained for the long term after the crush injury, concomitant with axon regeneration up to dLGN and SC, as well as synaptic reconnection at the SC.

## Material and methods

### Ethical considerations

All procedures involving human-derived materials were approved by and followed the guidelines of the Institutional Human Ethical and Research Committee of the Clementino Fraga Filho Hospital of the Federal University of Rio de Janeiro. Umbilical cords were donated after the mothers signed informed consent forms, as described in previous studies [41–44]. Experiments with animals followed the US National Institutes of Health guidelines and were approved and monitored by the Institutional Animal Care and Use Committee of the Federal University of Rio de Janeiro.

### hWJ-MSC isolation and cultures

Umbilical cords were collected in 200 mL of phosphate-buffered saline (PBS) with 1% antibiotics and fungizone (100 U/mL penicillin, 100 μg/mL streptomycin, amphotericin B, 250 g/mL; Life Technologies) at 4 °C. hWJ-MSC were isolated as previously described [42, 43]. Small pieces of isolated Wharton’s jelly were cut and digested for 16 h with collagenase type II (200 U/mL; Gibco, CA, USA) diluted in 100 mL Dulbecco’s modified Eagle’s medium F-12 (DMEM F-12; Gibco) with 1% antibiotics at 37 °C, under slow agitation. The digested material was washed in PBS, and the cell pellet was resuspended and plated in 75-cm^2^ plastic culture flasks in DMEM-F12 medium supplemented with 15% fetal bovine serum (FBS; Gibco) and 1% of both penicillin/streptomycin (Gibco). Cells were maintained with 5% CO_2_ in atmospheric air at 37 °C. After 3–5 passages, the cultures were highly enriched in hWJ-MSCs and were characterized by the expression of surface markers and differentiation potential as adipogenic or chondrogenic lineages [43].

To evaluate if serum deprivation (SD–) affects the therapeutic potential of hWJ-MSCs, the medium was withdrawn, the culture flasks were washed three times with PBS, and hWJ-MSCs were incubated with serum-free DMEM-F12 and antibiotics for 24 h. For therapy, cells in both conditions were detached at 80–90% confluence, using trypsin-EDTA solution (0.25% trypsin and 1 mM EDTA, Gibco). The trypsin was inactivated with DMEM-F12 medium containing 15% FBS, and the contents were washed three times with PBS by centrifuging for 5 min at 300×*g*, and the last time with Pulmozyme (recombinant human DNase I; 0.6 μL/mL; Roche). Cell viability was assessed by trypan blue staining. A total of 5 × 10^5^ hWJ-MSC or SD-hWJ-MSC was resuspended in 5 μL of sterile PBS containing Pulmozyme for intravitreal injection.

### Isolation of hWJ-MSCs-derived EVs

EVs secreted by hWJ-MSCs after 24 h in culture in serum-free medium were isolated as described previously [43, 45]. The medium was collected and sequentially centrifuged at 2000×*g* for 20 min and 100,000×*g* for 2 h at 4 °C (Optima L-90 K ultracentrifuge; Beckman Coulter), and the pellet was resuspended in PBS. Our group has previously found that hWJ-MSCs-derived EVs include microvesicles (100–1000 nm) expressing CD70, CD90, and CD105, and not expressing CD46, HLA-DR, and hematopoietic markers, and exosomes (30–150 nm) expressing CD63, CD9, and CD81 [43]. A quantity of 8.65 × 10^9^ EVs in 5 μL of PBS was aliquoted for injection, approximately equivalent to the quantity produced by 5 × 10^5^ hWJ-MSCs in 24 h [43].

### Optic nerve crush and intravitreal injection

Lister hooded rats (3 to 5 months old), with a mean weight of 200 g (females) and 300 g (males), were used in this study. Animals were housed with access to water and food ad libitum in a 12-h light/dark cycle, with all the procedures intended to minimize the number of animals used and their suffering. Animals were anesthetized with ketamine (75 mg/kg) and xylazine (5 mg/kg) injected intraperitoneally, and lidocaine was applied topically in the surgical region. Optic nerve crush was performed as previously described [8], briefly, at 1 mm behind the optic disc of the left eye with a forceps pressed on the optic nerve for 15 s, avoiding the ophthalmic artery and vein. Immediately after crush, a 5-μL suspension of 5 × 10^5^ hWJ-MSCs or SD-hWJ-MSCs, 8.65 × 10^9^ EVs, or vehicle (PBS + DNase) was injected into the vitreous body using a 5-μL Hamilton syringe at the limbus, avoiding injury to the lens. Before the injection, the same volume (5 μL) was aspired in order to avoid an increase in the intraocular pressure. After surgery, the skin was sutured and lidocaine ointment was applied. Animals were kept warm and under supervision until they recovered from the anesthesia. Animals with damage to the lens or blood vessels were excluded from the analysis.

### RGC survival analysis

To evaluate RGC survival after optic nerve crush, whole-mounted retinas were immunostained with Tuj1 antibody, which identifies βIII tubulin in retinal neurons and is widely used as an RGC marker [6, 11, 25, 46]. Animals were perfused through the heart with ice-cold saline, followed by 4% paraformaldehyde in 0.1-M phosphate buffer. Eyes were removed; retinas were dissected and washed 3 times with PBS with 0.5% Triton X-100 (Sigma; PBST-0.5%), followed by a 30-min incubation with 5% normal goat serum diluted in PBS. Retinas were then incubated with anti-β-III-tubulin antibody (Tuj1 mouse, Covance, 1:250) in 250 μL of PBST-2% for 18 h at 4 °C, washed with PBST-0.5%, and incubated with Alexa488-conjugated anti-mouse IgG produced in goat and ToPro-3 (both 1:1000, Life Technologies), in 250 μL of PBST-2% for 2 h at room temperature. Retinas were washed 3 times with PBS and then flat-mounted with 10% *p*-Phenylenediamine (PPD; 1 mg/mL in PBS) diluted in 90% glycerol. All washes and incubations were performed using a slow orbital shaker.

For quantification, 20 confocal images (each 0.05 mm^2^ in area) of Tuj1-stained retinas were obtained using a confocal microscope (one optical section 2.5 μm thick; × 40/0.8 Plan-NEOFLUAR oil-immersion objective; Zeiss LSM 510 Meta). Were generated images from 10 fields at 1 mm and 10 fields at 3.5 mm from the optic disc. The number of Tuj1^+^ cells was counted manually by a masked observer using Image J 5.2i (NIH, imagej.nih.gov, USA) and normalized by the number of RGCs in the contralateral retina. Statistical analysis was performed using a *t* test to compare two groups, or one-way ANOVA with Tukey’s multiple comparison test for more groups, in GraphPad Prism 6 (GraphPad Software Inc., San Diego, CA, USA).

### Axon staining and quantification

After perfusion, optic nerves and brains were dissected, cryopreserved in a sucrose gradient (10, 20 and 30%) in 0.1 M phosphate buffer (pH 7.4), and embedded in optimal cutting temperature medium (OCT, Tissue-Tek, Sakura, Japan). Optic nerves and brains were sectioned longitudinally and coronally on a cryostat (Leica, Wetzlar, Germany) at 14 and 20 μm thickness, respectively. Brain sections were collected from the optic chiasm to the posterior portion of the SC on gelatin-coated slides and stored at − 20 °C until immunostaining. For short-term analysis (14 days after crush), axons were labeled by immunostaining for GAP43 (1:50; Santa Cruz Biotechnology) for 18 h followed by a secondary Cy3-conjugated antibody (1:1000; Jackson ImmunoResearch) in 0.1% PBST. For long-term analysis (120 days after crush), axons were anterograde-labeled with Alexa 555-conjugated cholera toxin beta subunit (CTB; Life Technologies, 3 μL, 1:1 in sterile PBS) injected into the vitreous body 2 days before perfusion, according to protocols previously described [47].

Axons extending beyond the lesion site were counted at different distances from the injury site by a masked observer using a × 40/0.75 Plan-Neofluar objective (Axiovert 200 M microscope, Zeiss, Oberkochen, Germany). The total number of axons was estimated by the formula described by Leon and coworkers [48]. At least 3 sections were quantified for each animal. Statistical analysis was performed using two-way ANOVA for axonal extension analysis, in GraphPad Prism 6.

### Light deprivation and stimulation for *NGFI-A* quantification

To investigate whether the regenerated axons observed in the hWJ-MSCs-treated animals established synapses with SC neurons, we analyzed the expression of nerve growth factor-induced gene A (*NGFI-A*), a transcription factor expressed in SC neurons after visual stimulation [49]. To eliminate responses induced by the intact eye, the right optic nerve was transected 7 days before this experiment. The animals were anesthetized as described above, and the right optic nerve was cut with scissors.

To assess *NGFI-A* expression in the SC, the animals were light-deprived for 24 h, followed by 2 h of light stimulation before perfusion with paraformaldehyde. Tissue was prepared as described above. Brain sections were washed with PBST-0.1 blocked with normal goat serum and incubated overnight with a specific antibody against *NGFI-A* (anti-Egr1, 1:400, rabbit, Santa Cruz Biotechnology), followed by incubation with Alexa 488-conjugated goat anti-rabbit IgG antibody (1:1000) and ToPro-3 (1:1000; Life Technologies) and mounted with PPD. Images were acquired using a confocal microscope (Zeiss LSM 510 Meta), using a × 20/0.5 Plan-NEOFLUAR objective. Three brain sections on the rostro-caudal axis were chosen per animal, to obtain three images each of 0.135 mm^2^ from medial to lateral, covering the superficial layers of the SC. The number of *NGFI-A*^+^ cells was counted manually by a masked observer, normalized by the image area, and the data were separated into ipsilateral and contralateral sides of SC. Statistical analysis was performed using an unpaired parametric *t* test in GraphPad Prism 6 (GraphPad Software Inc., San Diego, CA, USA).

## Results

### hWJ-MSC treatment protects RGCs after optic nerve crush

**Fig. 1:**
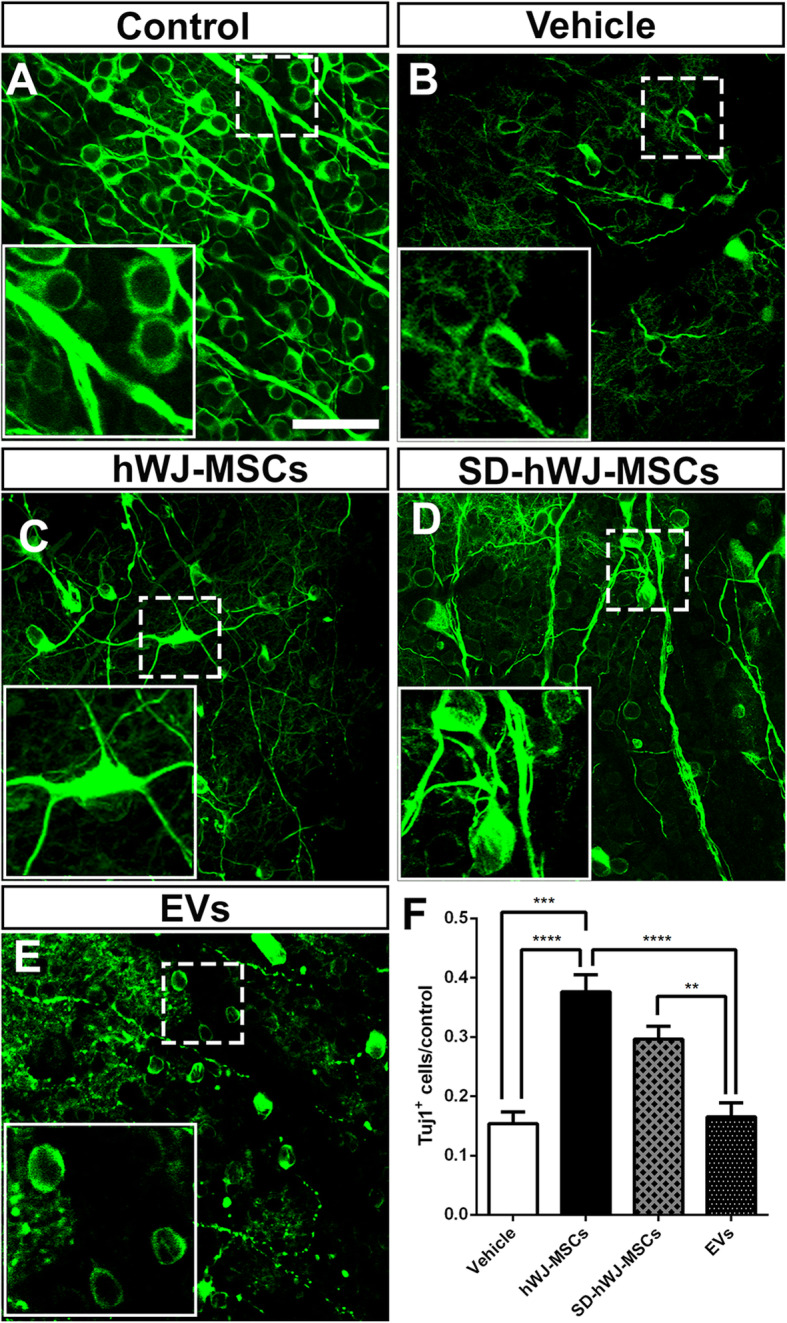
Survival of RGCs 14 days after crush and hWJ-MSC-based treatments. **a**–**e** Confocal images of an optical slice of whole-mounted retinas labeled for Tuj1, 14 days after crush and injection of vehicle, hWJ-MSCs, SD-hWJ-MSCs (after 24 h of fetal bovine serum deprivation), or EVs. Inset in the lower left corner of each image shows higher magnification of the dashed square. **f** Graph representing the number of RGCs of each experimental group normalized by control retinas. Mean ± SEM. One-way ANOVA with Tukey post-test, *P < 0.5, **P < 0.1, ***P < 0.001, ****P < 0.0001. Scale bars 50 μm for images and 21 μm for insets

Rodent MSCs have been reported to protect RGCs after optic nerve injuries [7]. However, validating the neuroprotective potential of human-derived MSCs is necessary before proceeding to clinical trials. Here, we found that 14 days after optic nerve crush and vehicle injection, the number of Tuj1^+^ retinal neurons was dramatically reduced to 15.39% of the number in the control retina (1142 ± 49.46 Tuj1^+^ cells/mm^2^ in control retinas; 117.8 ± 22.34 Tuj1^+^ cells/mm^2^ in the vehicle group; Fig. 1A, B, F), consistent with previous studies [5, 6, 8, 31, 50]. Treatment with hWJ-MSCs significantly increased RGC survival to 37.62% of the number in the control retina (429 ± 33.01 Tuj1^+^ cells/mm^2^ in the hWJ-MSC group; Fig. 1C, F). Since clinical protocols require that cell therapy be performed in xeno-free conditions [51], we evaluated if serum deprivation affects the neuroprotective potential of hWJ-MSCs. We found that there was no statistically significant effect of the presence or absence of serum on the neuroprotective effect, although the mean number of surviving RGCs treated with hWJ-MSCs cultured in the presence of serum was higher compared to SD-hWJ-MSCs (338.5 ± 25.00 Tuj1^+^ cells/mm^2^; Fig. 1D, F). For consistency, we used cells cultured in the presence of serum in the remaining experiments. In addition, we tested if the therapeutic effect of hWJ-MSCs could be reproduced by injecting only the EVs derived from these cells. The number of surviving RGCs 14 days after crush and treatment with EVs (188.4 ± 27.11 Tuj1^+^ cells/mm^2^) was similar to the vehicle-treated group, suggesting that EVs alone did not reproduce the neuroprotective effects of hWJ-MSCs, at least in the dose used in this study (Fig. 1E, F).

### Treatment with hWJ-MSCs provided sustained RGC neuroprotection

**Fig. 2:**
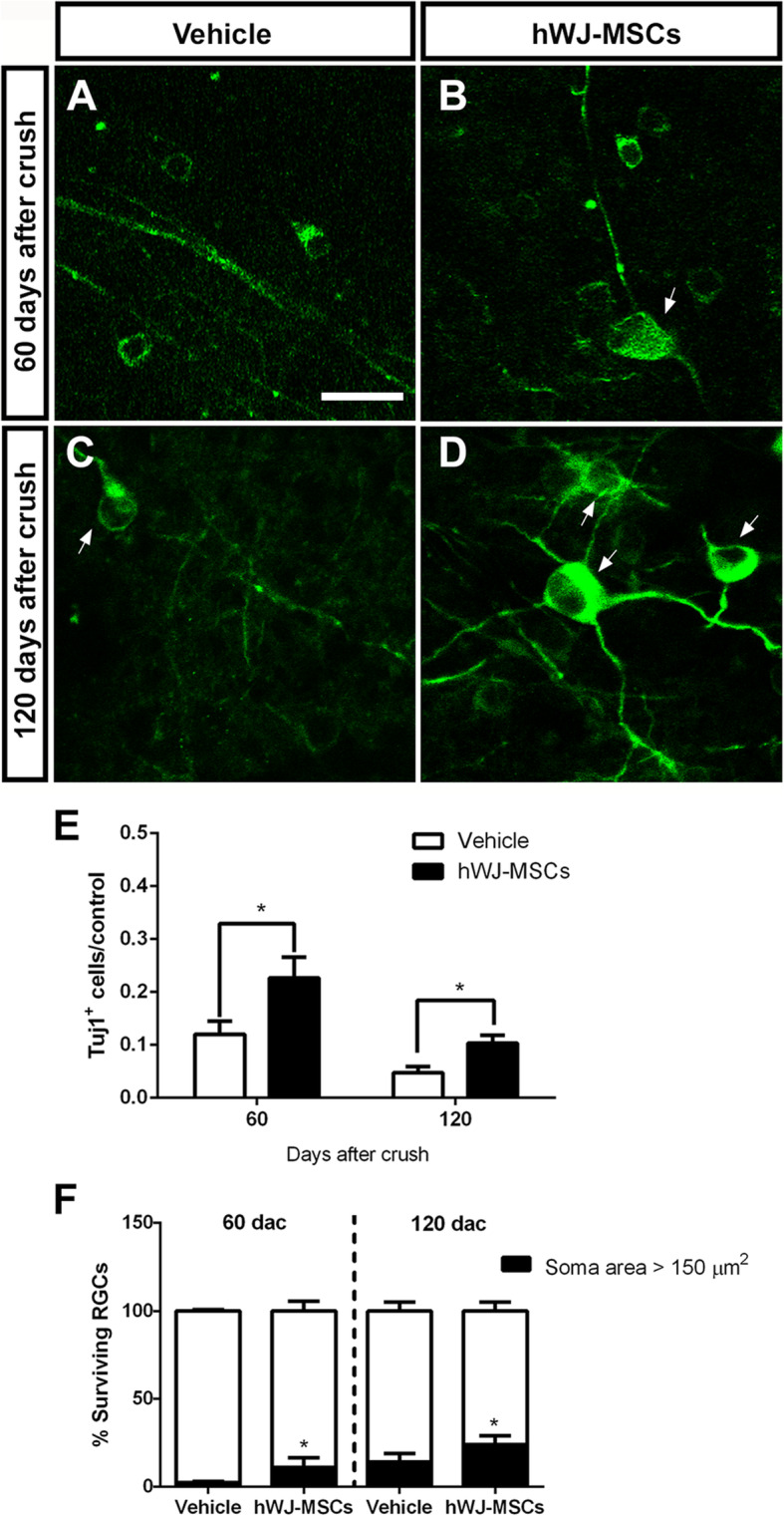
Long-term neuroprotective effect of hWJ-MSCs on RGCs. High magnification of confocal images of retinas immunostained for Tuj1 (**a**–**d**) 60 and 120 days after crush and injection of vehicle (**a** and **b**) or hWJ-MSCs (**c** and **d**). Arrows indicate RGCs with soma larger than 150 μm^2^. **e** Number of RGCs in the ipsilateral retina normalized by the contralateral eye. **f** Percentage of surviving RGCs with soma larger than 150 μm^2^ (black bars) at 60 and 120 days after crush and injection of vehicle or hWJ-MSCs. Mean ± SEM. Unpaired parametric *t* test, *P < 0.5. Scale bar 25 μm

To best assess the therapeutic potential, we investigated if the neuroprotective effect of hWJ-MSCs was sustained for periods longer than 14 days. At 60 and 120 days after crush, the number of Tuj1^+^ cells decreased to 10% and 5% of the control, respectively, in the vehicle-treated group (117.3 ± 28.94 Tuj1^+^ cells/mm^2^; and 49.15 ± 12.68 Tuj1^+^ cells/mm^2^; Fig. 2A–E). Notably, treatment with hWJ-MSCs led to a significant ~ 2-fold increase in the percentage of surviving RGCs at both time points (242.2 ± 42.16 Tuj1^+^ cells/mm^2^ or 22.63% survival at 60 days; and 107.6 ± 15.59 Tuj1^+^ cells/mm^2^ or 10.36% survival 120 days after crush; Fig. 2A–E).

### Treatment with hWJ-MSCs protects different subpopulations of RGCs

RGCs can differ according to their morphology, gene expression profile and electrophysiological properties [52]. Previous studies from our group showed that 10% of the total Tuj1^+^ cell population in rat retinas have large cell bodies (more than 150 μm^2^ of the soma size), cytoplasm densely labeled with Tuj1 and extensively arborized dendrites, and express osteopontin [31], which is consistent with the mouse αRGC subtype that has been reported to be more resilient to injury [53, 54]. We investigated whether treatment with hWJ-MSCs preferentially protected large RGCs at 60 and 120 days after optic nerve crush.

At 60 days after crush, we observed a very small population of Tuj1^+^ cells with soma size larger than 150 μm^2^ in the vehicle group (141.6 ± 26.5 Tuj1^+^ cells/retina, or 2.35% of the surviving RGCs). However, treatment with hWJ-MSCs protected significantly more large Tuj1^+^ cells (1266 ± 386.8 Tuj1^+^ cells/retina, or 11.02% of the surviving Tuj1^+^ cells) than in the vehicle group (*P* < 0.05). At 120 days after crush, we still found a significant difference in the number of large Tuj1^+^ cells after treatment with hWJ-MSCs (1648.65 ± 395 Tuj1^+^ cells/retina, or 24.05% of surviving Tuj1^+^ cells) in comparison to the vehicle group (394.8 ± 139.1 Tuj1^+^ cells/retina, or 14.14% of surviving Tuj1^+^ cells). The number of Tuj1^+^ cells smaller than 150 μm^2^ did not differ significantly between the vehicle and hWJ-MSCs, either 60 or 120 days after crush. Moreover, the number of smaller Tuj1^+^ cells decreased between 60 and 120 days in both groups, suggesting that they continued to degenerate. Interestingly, between 60 and 120 days after crush, in the hWJ-MSC-treated group, there was no significant decrease in RGCs larger than 150 μm^2^, suggesting that the cell therapy preferentially sustained the survival of large Tuj1^+^ cells, including the αRGCs. In addition to being more resistant to injury, αRGCs are known to extend axons after crush, mainly after mTOR pathway activation, and to re-grow axonal projections up to the SC [53].

### Treatment with hWJ-MSCs promotes RGC axonal regeneration to brain targets

**Fig. 3:**
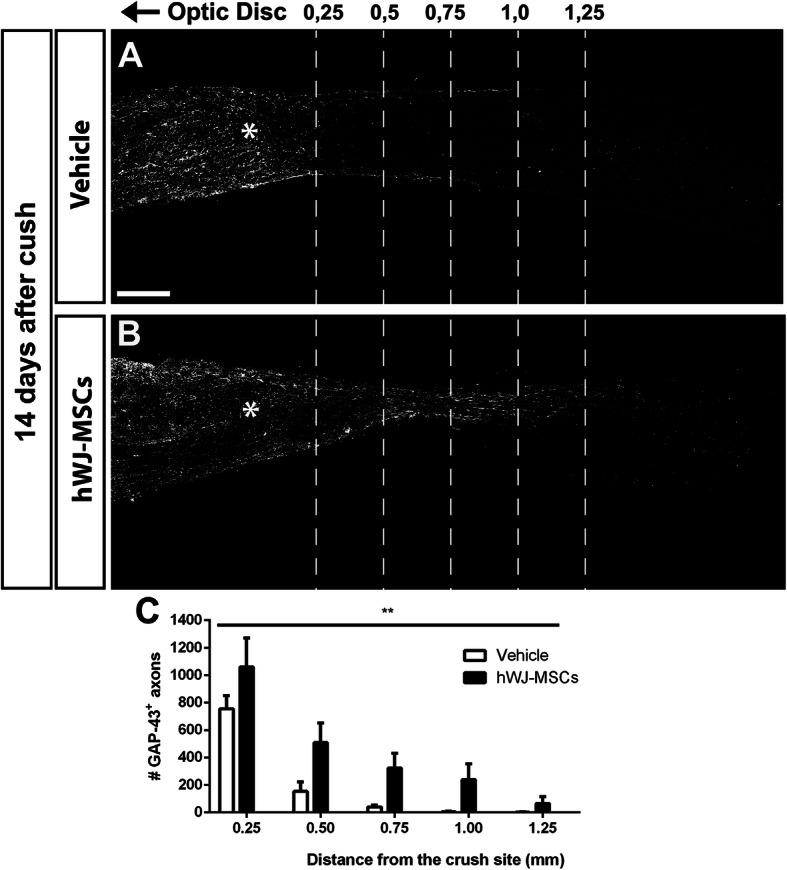
hWJ-MSC treatment promotes optic nerve regeneration. Photomontage of optic nerve sections immunostained for GAP-43 14 days after lesion and treatment with vehicle (**a**) or hWJ-MSCs (**b**). **c** Graph representing the mean ± SEM of axons at respective distances from the crush site in vehicle or hWJ-MSC-treated groups. Two-way ANOVA, **P < 0.01. Scale bar 200 μm

Because hWJ-MSCs provided a long-term neuroprotective effect, favoring survival of large RGCs, we investigated if the hWJ-MSC treatment promoted axonal outgrowth beyond the lesion site. For short-term analysis (14 days after crush), axonal regeneration was evaluated using the expression of GAP43, a protein expressed only by neurons during the axon growth process [15] (Fig. 3). Optic nerve crush led to GAP43 expression as observed in the vehicle group, yet we found only a few axons crossing the lesion site (Fig. 3A, C). Notably, the hWJ-MSC treatment increased the number of GAP43^+^ axons traveling over longer distances beyond the lesion site (Fig. 3B, C).

**Fig. 4:**
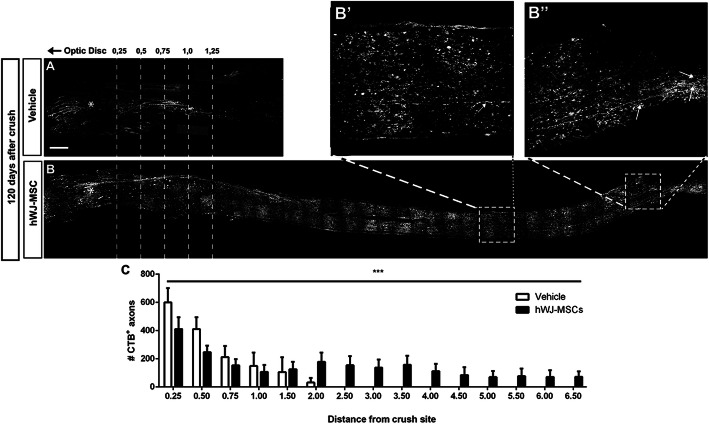
hWJ-MSC treatment promotes long-term axonal regeneration. Photomontage of optic nerve sections labeled with CTB-Alexa555 120 days after lesion and treatment with vehicle (**a**) or hWJ-MSCs (**b**). **b**′, **b**″ Insets of **b** with arrow indicating individual axons. **c** Graph representing the mean ± SEM of axons at respective distances from crush site in the vehicle- or hWJ-MSC-treated groups. Two-way ANOVA, ***P < 0.001. Scale bars 200 μm for **a** and **b**; 40 μm for **b**′ and **b**″

For long-term analysis, axons were anterogradely traced by intraocular injection of CTB. In the vehicle group, only a few axons were found up to 2 mm beyond the lesion site at 120 days after the crush (Fig. 4A, C). Treatment with hWJ-MSCs dramatically increased the number of labeled axons extending beyond the injury site at all time points compared to the vehicle, and 5 of 11 animals showed axons extending to the optic chiasm (approximately 6.5 mm from the lesion site, Fig. 4B, C).

**Fig. 5:**
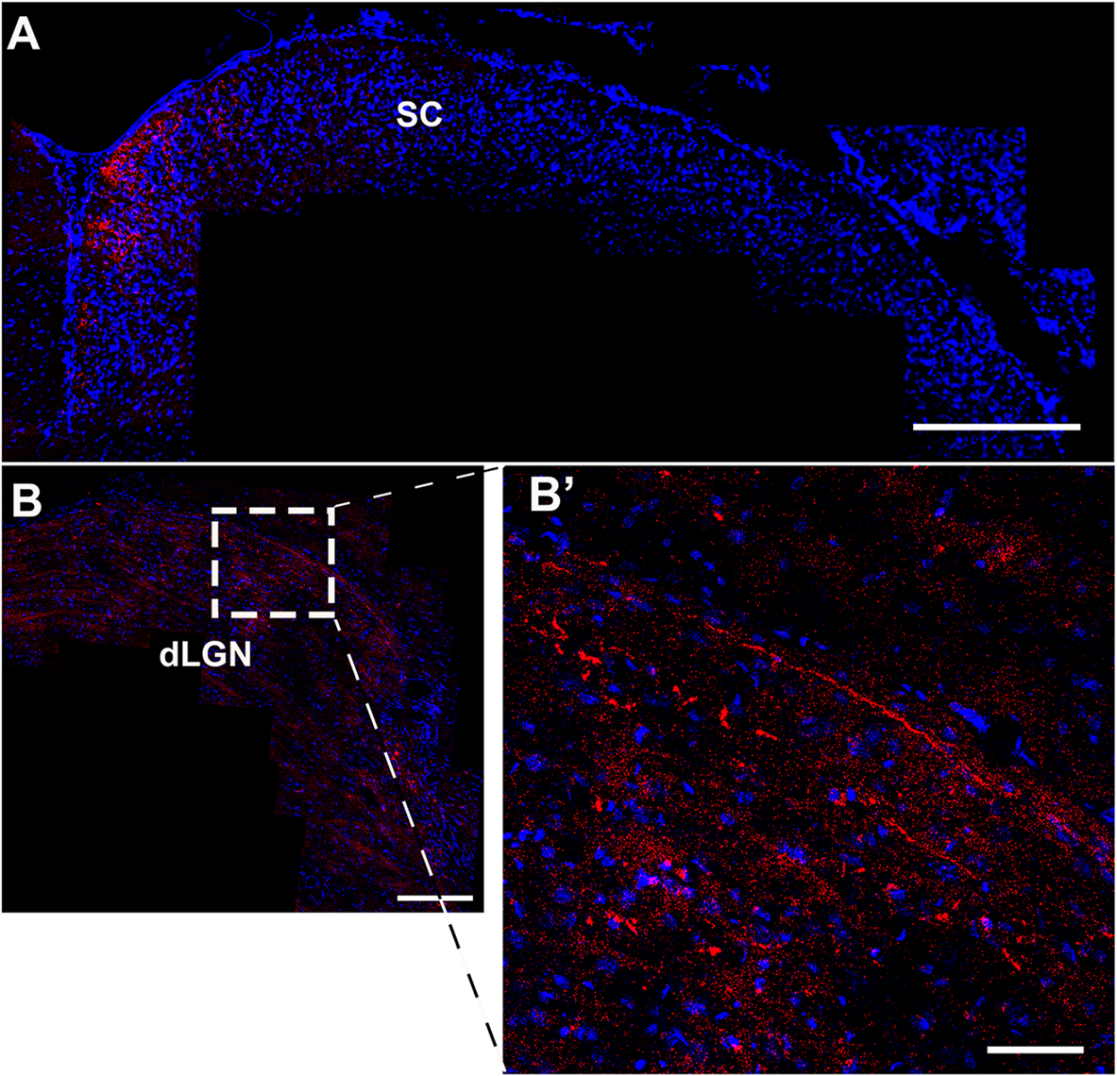
hWJ-MSC treatment promotes axonal regeneration up to the central nuclei. Representative photomontages of × 20 z-stack confocal images of coronal sections of the SC (**a**) and dLGN (**b** and **b**′) 120 days after optic nerve crush and injection of hWJ-MSCs. CTB labeling of the axonal terminals (red) and ToPro-3 nuclei (blue). Scale bars 200 μm (**A**); 25 μm (**a**–**b**′); 100 μm (**b**)

We also investigated whether these extending axons were able to reach the visual targets in the brain, by evaluation of coronal sections of the dLGN and SC (Fig. 5). Notably, we found regenerated RGC axons labeled with CTB in the dLGN (2 of 11 animals) and in the SC (4 of 11) at 120 days after optic nerve crush in animals treated with hWJ-MSCs (Fig. 5), suggesting that some axons could reach central targets. No CTB staining was found in brain sections of the vehicle group (*N* = 6), indicating absence of target reconnection in these animals. Therefore, treatment with hWJ-MSCs induced a marked RGC axon regrowth up to central targets.

### Treatment with hWJ-MSCs increases synaptic connection in the SC

**Fig. 6:**
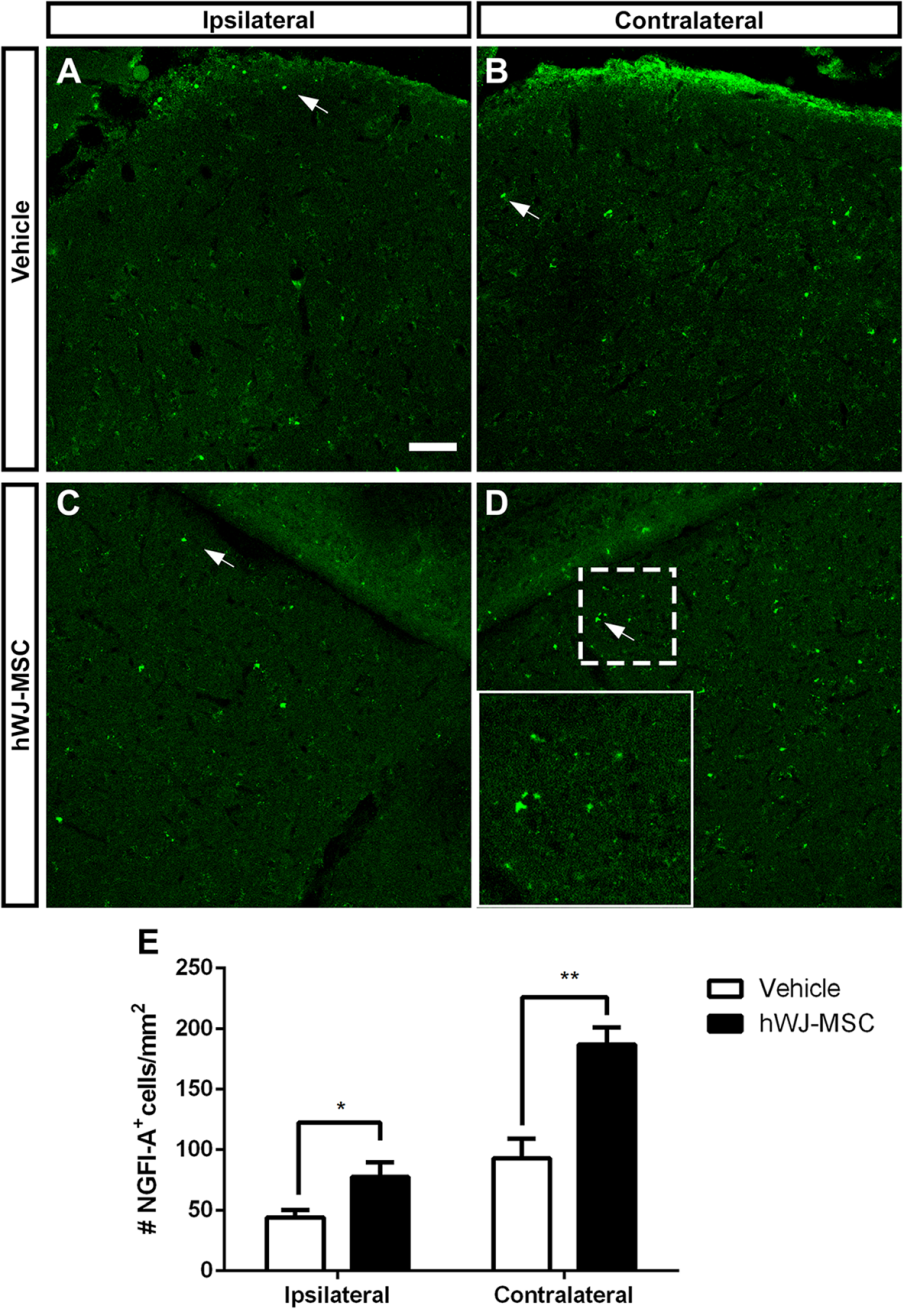
RGC terminals activate SC neurons. Images of × 20 z-stack confocal images. *NGFI-A* immunolabeling to identify activation of SC cells after light exposure in coronal section of ipsi- and contralateral SC 120 days after optic nerve crush and treatment with vehicle (**a**, **b**) or hWJ-MSCs (**c**, **d**). Inset in **d** shows higher magnification of the dashed square. **e** Quantification of the number of cells expressing *NGFI-A* per mm^2^ in each group ± SEM. Unpaired parametric *t* test, *P < 0.05, **P < 0.01. Scale bars 50 μm for images **a**–**d**; 23 μm for inset

To investigate if RGC axon regeneration and reinnervation led to formation of new synapses within brain targets, we analyzed the expression of light-induced *NGFI-A* in the SC. *NGFI-A* is an immediate early gene correlated with plasticity processes during development or after lesions [49, 55, 56]. *NGFI-A* expression is almost completely abolished in the SC after dark adaptation, and is upregulated after light exposure, in a mechanism dependent on glutamate released by retinal terminals binding to NMDA receptors in collicular neurons [49]. To investigate whether the regenerating axons reconnected to SC neurons, we quantified the number of cells expressing *NGFI-A* after dark adaptation and light stimulation. In the vehicle group, only a few *NGFI-A* expressing cells were found in both ipsi- and contralateral SC (44.35 ± 6.15 and 93 ± 16.23 *NGFI-A*^+^ cells/mm^2^, respectively; Fig. 6A, B, E). Since the right optic nerve was sectioned, and no axons were found beyond the lesion site in the left nerve, the *NGFI-A* expression in SC of the vehicle group was likely due to a basal expression of this factor. Treatment with hWJ-MSCs significantly increased the number of *NFGI-A*-positive cells both in the contralateral and ipsilateral SC, compared to the vehicle (77.52 ± 11.96 and 186.9 ± 14.34 *NGFI-A*^+^ cells/mm^2^, respectively; Fig. 6C, D, E), consistent with target reinnervation and synapse formation. This result suggests that, in addition to the neuroprotective and pro-regenerative stimulation of RGC axons up to the central targets in the brain, hWJ-MSCs transplantation promoted axon-target reconnection through glutamatergic synapses. However, despite this long-distance axonal regeneration up to visual targets (the dLGN and SC) and synapse reconnection, we have not observed functional recovery of visual behaviors (optokinetic or looming reflexes and dark/light preference test, data not shown).

## Discussion

Loss of RGCs is irreversible and can lead to partial or complete blindness. To date, no clinical therapy is available to protect RGCs and/or promote their axonal regrowth and target reconnection in the visual pathway. We investigated the neuroprotective and regenerative effects of intravitreal transplantation of hWJ-MSCs after optic nerve crush, which is a traumatic axonal injury leading to severe loss of RGCs [6, 8, 30, 50]. Treatment with hWJ-MSCs resulted in sustained survival of RGCs, marked long-distance axonal regeneration up to central targets, and partial recovery of the synaptic function.

MSCs can be obtained from several sources, including the human umbilical cord. This source is of particular interest because it is feasible to obtain and has advantageous biological properties. For instance, since the umbilical cord is obtained from newborns, it should have fewer epigenetic alterations than adult tissues [36], providing more consistent results when used for therapeutic purposes. Furthermore, the umbilical cord is a tissue necessarily collected at childbirth that is usually discarded and therefore does not require invasive procedures [36]. Compared to MSCs from other sources, for instance bone marrow, hWJ-MSCs can provide major trophic support and produce cytokines, including IL6, IL8, BDNF, LIF, NT-3, TGFβ2, and FGF2 [37, 57]. Importantly, MSCs from multiple sources do not show potential to generate tumors and teratoma, and compared to MSCs derived from human umbilical cord blood (hUCB-MSC), hWJ-MSCs express a wider range of tumor suppressor factors [37].

MSCs can be delivery through several routes. Here, we used the intravitreal approach, which has been shown to be neuroprotective for RGCs in several pre-clinical studies [1–6]. However, a few clinical studies using the route reported adverse effects [7, 8]. On the other hand, a combination of intravitreal, retrobulbar, subtenon, and intravenous injection of BMMSCs has been used in other studies with good safety and efficacy results [9–14]. Therefore, it is important to explore and/or combine other delivery routes to optimize cell therapies for retinal and optic nerve injuries. Both subtenon and retrobulbar injections are periocular routes that can be used to deliver molecules to the posterior segment of the eye and avoid the adverse effects of intravitreal injection [15]. Recently, a phase-3 clinical trial showed that a subtenon injection of hWJ-MSCs results in good outcomes for patients with retinitis pigmentosa [16]. This approach should be investigated in the optic nerve injury model and compared with other routes. In addition, a more direct approach such as a direct injection into the optic nerve should also be explored as recently described by Mesentier-Louro and colleges [17, 31]. In summary, safety and efficacy of different routes can be assessed in pre-clinical models in order to subsidize better clinical studies.

The effects of MSCs have been attributed to paracrine mechanisms rather than to differentiation into neural cells or integration into the retina [58]. Both direct release and/or delivery of EVs carrying molecules that act to promote survival or growth of the target cell have been suggested [39, 59]. In our experiments, we found that injection of hWJ-MSC-derived EVs did not protect RGCs. We injected a single dose of EVs released by the hWJ-MSCs over a period of 24 h in vitro, which could explain the negative results. MSCs survive in the vitreous body for several weeks and continuously release EVs. In addition, MSCs change the content of EVs released according to changes in the host tissue. All these factors could explain the absence of effects of EVs in our experiments and the positive results in other studies [60]. At this point, it is not possible to exclude the possibility that the beneficial effects of hWJ-MSC therapies are mediated by VE release. We also found that hWJ-MSCs exert neuroprotective effects independently of the presence of serum in the medium. Indeed, although MSC gene expression was altered after serum deprivation, this was not sufficient to interfere with their surface antigen expression, multipotentiality, and immunosuppressive potential [61]. These results support a neuroprotective effect of hWJ-MSCs cultured in xeno-free conditions, which has important implications for their safe clinical use.

Further, we found that treatment with hWJ-MSCs preferentially increased the survival of large-sized RGCs. While smaller RGCs continued to decay after crush, the number of larger RGCs was sustained over time in the hWJ-MSC-treated group compared to the vehicle-treated group. It has been estimated that more than 46 different subtypes of RGCs are found in the rodent retina, differing in their morphology, localization, gene expression, and physiological properties [54]. As Tuj1^+^ cells with more than 150 μm^2^ area comprise mostly the αRGCs [31], and this RGC type is known to project axons to the SC [62], it is possible that the treatment selectively targeted αRGCs. Indeed, it has been shown that αRGCs regenerate their axons after activation of the mTOR pathway and are among the most resistant RGC subtypes after optic nerve lesion [53, 54, 62].

In addition to the survival of RGCs, we also found marked axonal regeneration in animals treated with hWJ-MSCs. While none of the animals in the vehicle group had axons regenerating farther than 2 mm from the lesion site, we observed staining for CTB in the optic chiasm of 5 out of 11 animals, and in the SC of 4 out of 11 animals in the hWJ-MSC-treated group, at 120 days after crush, showing that the treatment promoted the regeneration of RGC axons to these targets. Notably, we demonstrated evidence of the reconnection of RGCs with the SC through the re-establishment of active glutamatergic synapses, as shown by increased *NGFI-A* expression. The small number of *NGFI-A* expressing cells in SC of both the ipsi- and contralateral sides in the vehicle group was likely a result of a basal expression of *NGFI-A*, and all animals were subjected to transection of the non-crushed nerve 7 days before the light-stimulation experiment to eliminate a light-response from the uncrushed visual pathway. In addition, some evidence indicates that the absence of vision induces plasticity in subcortical visual areas, which can receive stimulus from areas related to hearing [63].

As well as the allogenic therapy with bone marrow-derived MSCs (BM-MSCs) [6, 31], treatment with hWJ-MSCs provided a long-term effect capable of delaying the death of RGCs and promote axonal outgrowth for long distances in the optic nerve, leading to target reconnection in nearly half of the animals that were treated. Although several studies have described therapeutic effects of human MSCs after optic nerve lesion [47, 58, 64, 65], none has investigated their long-term effect or demonstrated target reconnection. For example, our results differ from those of other studies using hUCB-MSCs and hWJ-MSCs, which found a loss of the neuroprotective effect after 28 days [66, 67]. The sustained neuroprotective and pro-regenerative effects that we observed could be explained by the dose used here, which was 25 times higher than that used by Millán-Rivero [67], revealing the importance of dose studies in the pre-clinical setting.

To the best of our knowledge, only a few approaches have been able to promote axonal regeneration to brain targets after optic nerve injury. Our group previously demonstrated that RGC axons can regenerate to the SC after therapy with BMMCs in an optic nerve crush model [8]. Umbilical cord-derived MSCs have been shown to promote axonal regeneration and protect RGCs in an optic tract lesion model [68]. It has also been shown that in mice subjected to optic-nerve lesion, the induction of inflammation combined with cAMP injection and deletion of *PTEN* induces axons to regenerate to central targets [27]. It has been reported that activation of the mTOR pathway together with visual simulation to the injured eye leads to a striking regeneration, with axons extending to the suprachiasmatic nucleus, dLGN, pretectal nucleus, medial terminal nucleus, and SC [62]. This visual stimulation could be used in combination with the treatment with hWJ-MSCs described here to amplify the outgrowth capability of RGCs.

Axonal regeneration to visual targets is a challenge for optic nerve treatments, and many efforts have been made to develop regenerative strategies. The present study is the first to demonstrate a sustained protective and regenerative effect on rat adult axons after therapy with human-derived MSCs in an optic nerve lesion model. These findings highlight the effectiveness of the cell therapy using an easily obtained source of human MSCs for future clinical studies. However, many preclinical studies are still needed before translational studies can be performed.

## Conclusions

This study presents evidence of positive effects of cell therapy using hWJ-MSCs for optic nerve injury. We have shown the potential of allogenic transplantation of MSCs to protect and promote RGC axon outgrowth in the animal model used here and for the first time have shown a long-term effect of human-derived MSCs. Even 120 days after injury, the hWJ-MSC-treated group presents a higher number of RGCs and their axons reached and make synapses with the SC. This finding is useful for the development of therapeutic strategies, since the umbilical cord is a reliable source of MSCs, insofar as it is a highly available tissue that is usually discarded and can be collected without invasive procedures. Although the results are promising, additional data about cell therapy with hWJ-MSCs are needed to understand the neuroprotective and regenerative effects. Further steps should be taken to understand the cellular and molecular mechanisms by which hWJ-MSCs exert their effect and to develop combined approaches to enhance the therapeutic effect.

## Data Availability

The datasets used and/or analyzed during the current study are available from the corresponding author on reasonable request.

## Abbreviations

BMMCs: Bone marrow-derived mononuclear cells
BM-MSCs: Bone marrow-derived MSCs
CTB: Cholera toxin beta subunit
DMEM F-12: Dulbecco’s modified Eagle’s medium F-12
EVs: Extracellular vesicles
dLGN: Dorsal lateral geniculate nucleus
FBS: Fetal bovine serum
GAP43: Growth-associated protein 43
hUCB-MSCs: Human umbilical cord blood-derived MSCs
hWJ-MSCs: Human umbilical Wharton’s jelly-derived MSCs
MSCs: Mesenchymal stem cells
*NGFI-A*: Nerve growth factor-induced gene A
OCT: Optimal cutting temperature
PBS: Phosphate-buffered saline
PBST: PBS with Triton-X
PPD: p-Phenylenediamine
Pten: Phosphatase and tensin homolog
RGCs: Retinal ganglion cells
SC: Superior colliculus
SD–: Serum deprivation
Socs3: Suppressor of cytokine signaling 3
